# Distinguishing apathy from depression: A review differentiating the behavioral, neuroanatomic, and treatment‐related aspects of apathy from depression in neurocognitive disorders

**DOI:** 10.1002/gps.5882

**Published:** 2023-02-05

**Authors:** Krista L. Lanctôt, Zahinoor Ismail, Kritleen K. Bawa, Jeffrey L. Cummings, Masud Husain, Moyra E. Mortby, Philippe Robert

**Affiliations:** ^1^ Departments of Psychiatry and of Pharmacology and Toxicology University of Toronto Toronto Ontario Canada; ^2^ Neuropsychopharmacology Research Group Hurvitz Brain Sciences Program Sunnybrook Research Institute Toronto Ontario Canada; ^3^ Bernick Chair in Geriatric Psychopharmacology Sunnybrook Health Sciences Centre University of Toronto Toronto Ontario Canada; ^4^ Departments of Psychiatry, Clinical Neurosciences, and Community Health Sciences Hotchkiss Brain Institute O'Brien Institute of Public Health University of Calgary Calgary Alberta Canada; ^5^ Department of Brain Health Chambers‐Grundy Center for Transformative Neuroscience School of Integrated Health Sciences University of Nevada Las Vegas (UNLV) Las Vegas Nevada USA; ^6^ Nuffield Department of Clinical Neurosciences University of Oxford Oxford UK; ^7^ Department of Experimental Psychology University of Oxford Oxford UK; ^8^ School of Psychology University of New South Wales Sydney New South Wales Australia; ^9^ Neuroscience Research Australia Sydney New South Wales Australia; ^10^ Cognition Behaviour Technology Lab University Côte d'Azur (UCA) Nice France; ^11^ Centre Mémoire Le Centre Hospitalier Universitaire de Nice Nice France

**Keywords:** apathy, depression, diagnosis neuropsychiatric impairment, neurocognitive disorders

## Abstract

**Objectives:**

This narrative review describes the clinical features of apathy and depression in individuals with neurocognitive disorders (NCDs), with the goal of differentiating the two syndromes on the basis of clinical presentation, diagnostic criteria, neuropathological features, and contrasting responses to treatments.

**Methods:**

Literature was identified using PubMed, with search terms to capture medical conditions of interest; additional references were also included based on our collective experience and knowledge of the literature.

**Results:**

Evidence from current literature supports the distinction between the two disorders; apathy and depression occur with varying prevalence in individuals with NCDs, pose different risks of progression to dementia, and have distinct, if overlapping, neurobiological underpinnings. Although apathy is a distinct neuropsychiatric syndrome, distinguishing apathy from depression can be challenging, as both conditions may occur concurrently and share several overlapping features. Apathy is associated with unfavorable outcomes, especially those with neurodegenerative etiologies (e.g., Alzheimer's disease) and is associated with an increased burden for both patients and caregivers. Diagnosing apathy is important not only to serve as the basis for appropriate treatment, but also for the development of novel targeted interventions for this condition. Although there are currently no approved pharmacologic treatments for apathy, the research described in this review supports apathy as a distinct neuropsychiatric condition that warrants specific treatments aimed at alleviating patient disability.

**Conclusions:**

Despite differences between these disorders, both apathy and depression pose significant challenges to patients, their families, and caregivers; better diagnostics are needed to develop more tailored treatment and support.

## Key points

- Apathy and depression are distinct neuropsychiatric syndromes that share overlapping features and may occur concurrently in individuals with neurocognitive disorders (NCDs)
- Distinguishing apathy from depression can be challenging, but proper identification of each syndrome is important for appropriate intervention
- This narrative review describes clinical features associated with apathy and depression and differentiates each syndrome based on clinical presentation and diagnostic criteria, neuropathological features, and differences in response to treatment

## 1 INTRODUCTION

Apathy, a frequently occurring neuropsychiatric aspect of neurodegenerative disease, is characterized by symptoms of diminished initiative, decreased interest, and/or impaired emotional expression/responsiveness.^1^ Per the most recent diagnostic criteria, syndromic apathy persists or recurs for ≥4 weeks, represents a change from the patient's usual behavior, causes significant functional impairment, and is not exclusively explained by other etiologies.^1^ As the world population ages, the prevalence of neurodegenerative conditions will concurrently increase,^2^ and therefore so will apathy. Recognizing apathy as a dementia‐related syndrome is critical to the optimal care of individuals with neurocognitive disorders (NCDs), their families, and their caregivers. Apathy is prevalent across NCDs, including Alzheimer's disease (AD; prevalence range, 24%–85%),^3^ frontotemporal dementia (FTD; 50%–100%),^4^ Huntington's disease (HD; 52%–76%),^5^ progressive supranuclear palsy (PSP; 20%–92%),^6^ Parkinson's disease (PD; 17%–70%),^7^ Lewy body dementia (35%–100%),^8^ vascular dementia (VaD; 43%–89%),^3^ and mild cognitive impairment (MCI; 10.7%–44.8%).^9^

Depression is also common in NCDs and in a clinical setting, apathy can be difficult to differentiate from depression because these neuropsychiatric syndromes often occur concurrently and have several overlapping symptoms.^10^ Previous research has demonstrated that apathy and depression co‐occur in dementia and pre‐dementia states, with co‐occurrence estimated in 14%–38% of individuals with NCDs.^11^, ^12^ Because both apathy and depression negatively impact patient and caregiver quality of life and disease prognosis, addressing under‐recognition, conflation, or poor differentiation of these conditions is important to ensure appropriate or effective treatments to reduce symptom burden.^13^, ^14^, ^15^, ^16^, ^17^, ^18^, ^19^, ^20^

Here we review the literature on apathy and depression in NCDs. Literature was identified using PubMed, with search terms to capture medical conditions of interest (e.g., ‘neurocognitive disorders’, ‘apathy’, ‘depression’); additional references were also included based on our collective experience and knowledge of the literature. We were guided methodologically by the Scale for the Assessment of Narrative Review Articles, which advocates for the development of high‐quality narrative reviews based on key criteria, including a justification of the article's importance, statement of specific aims to guide development, referencing, and scientific reasoning.^21^

In this review, we aim to describe both overlap between these syndromes and evidence supporting their differentiation, including clinical presentation and symptoms, diagnostic criteria, potential differences in neurobiological characteristics, and contrasting responses to treatments. Although apathy occurs across a range of conditions,^22^ this narrative review focuses solely on NCDs, defined by the Diagnostic and Statistical Manual of Mental Disorders Fifth Edition (DSM‐5) as disorders in which impairment of cognitive function is the primary clinical deficit and represents a decline from the individual's prior level of function.^23^

## 2 DEFINITIONS AND CLINICAL PRESENTATION OF APATHY AND DEPRESSION

Apathy was first defined by Marin over 30 years ago as a disorder of diminished motivation not attributable to diminished levels of consciousness, cognitive impairment, or emotional distress.^1^, ^24^ Over time, various definitions of apathy have been proposed,^1^, ^25^, ^26^, ^27^, ^28^, ^29^ describing apathy as a disorder of self‐initiation,^29^ diminished goal‐directed behavior or cognition,^28^ and lack of initiative or novelty‐seeking behavior^27^ (a comprehensive review of the evolution of the definition of apathy can be found in Miller et al.^1^). Given these various descriptions, the International Society for CNS Clinical Trials and Methodology (ISCTM) Apathy Workgroup, a group of experts from academia, industry, and regulatory bodies, used a modified Delphi approach to develop consensus criteria for the diagnosis of apathy in NCDs.^1^ These criteria address primary diagnosis, symptom dimensions and symptom duration, exclusionary criteria, and severity. Specifically, patients must meet criteria for a syndrome of cognitive impairment or dementia and exhibit at least one symptom in at least two of the following dimensions: diminished initiative, diminished interest, and diminished emotional expression/responsiveness. Patients do not meet criteria for apathy if these symptoms can be better explained by other factors such as intellectual, physical, or motor disability, changes in level of consciousness, or the direct effect of substance use. Finally, to meet the criteria for apathy, these symptoms must be severe enough to produce impairment in personal, social, occupational, and/or other domains of function. These consensus criteria were recently published, and research and clinical practice have historically relied upon various other definitions or measures to identify apathy. Rating scales commonly used to evaluate apathy are summarized in Table 1.

**TABLE 1:** Summary of assessments used to evaluate apathy and depression.

Assessment	Comment
Apathy	
Apathy Evaluation Scale^30^	3 versions for self‐evaluation, clinician evaluation, and informant evaluation Evaluates apathy across the domains of overt behavior, cognitive aspects of motivation, and emotional responsivity Validated on a mixed sample of stroke, AD, depression, and community‐dwelling older adults
Apathy Inventory^26^	Oral interview with caregiver‐ and patient‐based versions Target population: MCI, PD, AD
Apathy Motivation Index^31^	Evaluates apathy across the domains of behavioral activation, emotional sensitivity, and social motivation Validated for use in individuals with alcohol use disorder and Korsakoff's syndrome,^32^ PD,^32^ and limbic encephalitis,^33^ and caregivers^33^
Dementia Apathy Interview Rating Scale^34^, ^35^	Structured interview conducted with the primary caregiver Evaluates behavior, interest, and engagement with the environment over the past 4 weeks using frequency of each behavior, compared with behavior before AD diagnosis
Dimensional Apathy Scale^36^	Evaluates emotional, executive, and cognitive/behavioral initiation dimensions of apathy Validated in AD, ALS, and PD^37^, ^38^, ^39^
Frontal Systems Behavior Scale^40^	3 subscales assess apathy, disinhibition, and executive dysfunction Suitable for use with patients who have damage to frontal‐subcortical brain circuits (e.g., TBI, AD, PD)
Geriatric Depression Scale (3 select items)^17^	Self‐rating scale Items include the following questions: ‐ Have you dropped many of your activities? ‐ Do you prefer staying at home, rather than going out and doing new things? ‐ Do you feel full of energy?
Interest Game^41^	An application‐based game to evaluate apathy in individuals with neurocognitive disorders Provides evaluation of patient interests across a variety of categories (e.g., eating, family, sports, reading)
Lille Apathy Rating Scale^27^	Clinician‐administered interview Initially validated in patients with PD^27^; additionally validated in early mild‐to‐moderate dementia^42^
Mild Behavioral Impairment Checklist—Apathy Domain^43^	Developed for use in pre‐dementia populations, but has also been used in dementia populations Questions inform on interest, initiative, and emotional reactivity Rated by family members/close informants or patient/participant
Neuropsychiatric Inventory‐Apathy Scale^25^	The NPI evaluates 12 behaviors with a series of scripted questions administered to the caregiver Validated on a sample of patients with AD, VaD, and other types of dementing disorders
Neuropsychiatric Inventory‐C Scale‐Apathy/Indifference^44^, ^45^	Adapted from original NPI scale to include clinician participation Some scales have been divided (i.e., agitation/aggression was split into separate agitation and aggression scales) and can be used as standalone scales
Neuropsychiatric Inventory‐Q Scale—Apathy/Indifference^45^, ^46^	Adapted from original NPI scale and includes only the screening questions without subquestions, the severity rating without frequency, and added caregiver distress Completed by caregiver
Depression	
Behavioral Pathology in AD Rating Scale (BEHAVE‐AD)^47^	Measures behavioral and psychological symptoms of dementia (including depressed mood) in AD Rating scale based on informant interview
Columbia University Scale for the Psychopathology of AD^48^	Clinician‐administered, semi‐structured instrument to rate symptoms of psychosis, behavioral disturbances, and depression in AD
Cornell Scale for Depression in Dementia^49^	Assesses the signs and symptoms of major depression in individuals with dementia Scored using two semi‐structured interviews given to the patient and an informant
Dementia Mood Assessment Scale^50^	Similar to the Hamilton Depression Scale, but does not include subjective elements to facilitate completion in patients with dementia For use in patients with mild‐to‐moderate dementia
Geriatric Depression Scale^51^	Screens for depression with a series of yes/no questions Suitable for use in physically healthy, physically ill, and cognitively impaired older adults
Hamilton Depression Rating Scale^52^	Evaluates depressive symptoms experienced over the past week Scores are obtained through unstructured or semi‐structured clinical interviews
Montgomery‐Åsberg Depression Rating Scale^53^	A 10‐item scale that evaluates the presence and severity of depressive symptoms through clinician interviews^53^ Validated in early‐onset dementia,^54^ AD,^55^ and PD^56^, ^57^ A proxy‐based version has additionally been validated as a screening tool for nursing home residents with dementia (including AD and VaD)^58^
Neuropsychiatric Inventory‐Depression Scale^25^	The NPI evaluates 12 behaviors with a series of scripted questions administered to the caregiver Validated on a sample of patients with AD, VaD, and other types of dementing disorders Depression scores are part of the NPI assessment
Neuropsychiatric Inventory‐C Scale—Dysphoria/Depression^44^, ^45^	Adapted from original NPI scale to include clinician participation Some scales have been divided (i.e., agitation/aggression was split into separate agitation and aggression scales) and can be used as standalone scales The depression/dysphoria domain of the NPI‐C scale can be used as a standalone measure
Neuropsychiatric Inventory‐Q Scale—Dysphoria/Depression^45^, ^46^	Adapted from original NPI scale and includes only the screening questions without subquestions, the severity rating without frequency, and added caregiver distress Completed by caregiver Depression is evaluated as one of the symptom domains

Abbreviations: AD, Alzheimer's dementia; ALS, amyotrophic lateral sclerosis; MCI, mild cognitive impairment; PD, Parkinson's disease; TBI, traumatic brain injury; VaD, vascular dementia.

Unlike apathy, depression has well‐defined diagnostic criteria presented in the DSM‐5, manifesting at least five of nine possible symptoms occurring every day or nearly every day over a 2‐week period. Symptoms include depressed mood and markedly diminished interest or pleasure in activities, which are cardinal symptoms—the presence of at least one of which is required to meet diagnostic criteria.^23^ Accessory symptoms include significant change in weight, insomnia or hypersomnia, psychomotor agitation or retardation, fatigue or loss of energy, feelings of worthlessness or excessive/inappropriate guilt, diminished ability to think or concentrate, or recurrent thoughts of death.^23^ In addition to these criteria, disease‐specific diagnostic criteria for depression occurring in AD and PD have been proposed to account for the unique presentation of depression in these circumstances.^59^, ^60^, ^61^ For example, disease‐specific provisional diagnostic criteria for depression in PD suggest evaluating whether mood disturbances are related to motor fluctuations with antiparkinsonian medications (i.e., ‘*on*’ and ‘*off*' states), with specific guidance for assessment during *on* states, and noting if mood fluctuates between *on*, *off*, or both states.^60^ For AD, provisional diagnostic criteria suggest a minimum of three depressive symptoms required for diagnosis, with specific guidance to exclude symptoms potentially related to non‐mood‐related characteristics of dementia (e.g., weight loss due to difficulties with food intake).^61^ Finally, along with symptoms outlined in the DSM‐5, symptoms of social isolation/withdrawal or irritability may be present. Rating scales have been developed to assist in standardized collection of relevant behavior and psychological information; disease‐specific and non‐disease‐specific clinical rating scales used for evaluating depression are described in Table 1.

### 2.1 Similarities between apathy and depression

#### 2.1.1 Clinical presentation

Apathy and depression are distinct clinical syndromes with several overlapping symptoms, including reduced interest and initiative, as well as decreased motivation.^10^ Anhedonia, defined in DSM‐5 as ‘markedly diminished interest or pleasure in all, or almost all, activities most of the day, nearly every day’ is a core symptom of major depressive disorders.^23^ As a result, rating scales for depression may contain apathy‐related items with the aim of capturing this cardinal symptom of depression.^11^, ^62^, ^63^ In an analysis evaluating the intercorrelation between items measured on the Apathy Evaluation Scale (AES) and the Hamilton Depression Scale (HAM‐D), two commonly used assessments of apathy and depression, items related to diminished work/interest, psychomotor retardation, energy, and lack of insight on the HAM‐D were found to be highly correlated with the AES total score.^62^ Similarly, the Geriatric Depression Scale (GDS) includes three items that have been validated to evaluate apathy (described in Table 1), but scores on these items contribute to overall ratings of depression.^17^, ^64^ Depression scales that include items related to apathy may lead to misclassification of patients and further overestimation of the overlap between each syndrome and underestimation of the prevalence of apathy.^11^

#### 2.1.2 Prognostic significance

Both apathy and depression consistently predict an increased risk for the development of NCDs. A meta‐analysis of 10 studies that included over 22,000 individuals classified as cognitively normal found that apathy was associated with a 2‐fold increased risk of cognitive impairment.^65^ Indeed, it has been suggested that apathy may be a pre‐dementia phenotype.^16^ In a study of 1008 community‐dwelling older adults with scores on the apathy domain of the Neuropsychiatric Inventory (NPI‐A) rating scale, of those who were defined as cognitively normal, apathy was associated with lower scores on several neuropsychologic tests. Similarly, a meta‐analysis of 16 studies comprising over 7000 patients recruited from memory clinics concluded that apathy was associated with a 2‐fold increased risk of progression to dementia.^66^ Across etiologies of NCDs (including PD, FTD, HD, AD), the prevalence of apathy increases over time and severity is associated with illness progression.^67^, ^68^, ^69^, ^70^, ^71^ These findings emphasize the importance of this neuropsychiatric syndrome on patient outcomes, suggesting that the presence of apathy may be indicative of more malignant form of disease.^72^

In individuals with a history of depression, a meta‐analysis of 20 case‐control and cohort studies showed a doubling of odds of development of dementia (odds ratio, 2.02; 95% confidence interval, 1.8–2.26).^73^ Another meta‐analysis of 18 studies that pooled data from 10,861 individuals with MCI found that the relative risk of progression to dementia was 28% higher in individuals with symptoms of depression compared with those without.^74^ Even when accounting for behavioral disturbances and dementia severity, depression is associated with transfer from assisted living to facilities providing a higher level of care.^75^

These studies suggest that apathy and depression have overlapping symptoms, with both syndromes having prognostic significance for development of dementia.

#### 2.1.3 Patient and caregiver burden

Both apathy and depression are associated with substantial burden for patients and their caregivers. In individuals with NCDs, apathy is associated with impairments in activities of daily living,^76^, ^77^, ^78^, ^79^ a consistent finding even when accounting for executive dysfunction,^78^ or for demographic factors and general cognitive status.^79^ As one of the most frequently lamented behaviors by caregivers, apathy in patients with dementia is associated with high levels of caregiver burden and distress.^13^, ^15^, ^80^, ^81^, ^82^ Caregivers often misinterpret apathy as oppositional or volitional behavior, and spouses of individuals with apathy report a loss of connection to the patient, which affects marital relationships.^83^ In a meta‐analysis of 16 studies reporting on the relationship between behavioral and psychological symptoms in dementia and caregiver burden, apathy was one of the top‐three most distressing symptoms, following depression and agitation/aggression.^15^ Interviews evaluating the experiences of caregivers of individuals with apathy and dementia found that apathy led to emotional and physical demands for caregivers; these demands were attributed to an unequal balance in roles and responsibilities at home, frustration with the lack of insight and awareness of apathy, and feelings of guilt over strategies they used to manage apathy.^13^

Depression is associated with functional dependence and caregiver burden in those with NCDs. In individuals with AD, those with major depression present with more severe non‐mood behavioral disturbances, higher rates of serious wandering, and greater dependency for self‐care compared with those with minor depression.^84^ Additionally, individuals with minor depression demonstrate significantly higher rates of wandering and more severe non‐mood behavioral disturbances compared to those with no depression.^84^ Finally, depression in individuals with dementia has been associated with increased caregiver burden,^85^ as well as higher rates of depression among live‐in caregivers compared with non‐live‐in carers.^86^

Taken together, these results suggest that apathy and depression may affect family and caregivers to different degrees, emphasizing the importance of better diagnostics to develop more tailored support mechanisms for these disorders.

## 3 DIFFERENTIATING APATHY FROM DEPRESSION ON THE BASIS OF DIAGNOSTIC CRITERIA AND CLINICAL FEATURES

Whereas apathy is considered a disorder characterized by a reduction in self‐initiated goal‐directed activities, depression has elements of sadness and/or anhedonia.^1^, ^23^ Although volition is predominantly affected in apathy, and mood is predominantly affected in depression, each syndrome may be further distinguished based on thought content, behavior, suicidality, anxiety, rumination, and the presence of vegetative symptoms.^10^

Studies that have included patients with apathy without depression, or depression without apathy, provide evidence that these two syndromes are distinct.^11^, ^12^, ^18^, ^87^, ^88^, ^89^, ^90^, ^91^ A number of studies investigating patients with both apathy and depression have further demonstrated distinctions between the two syndromes.^11^, ^70^, ^92^ In an investigation comparing apathy and depression across several NCDs (i.e., AD, FTD, PD, HD, and PSP), apathy and depression were not correlated across diagnostic subgroups and the presence of one syndrome did not predict the other.^11^ Furthermore, the authors reported that apathy (but not depression) was associated with aberrant motor behavior and disinhibition, whereas depression (but not apathy) was associated with anxiety, agitation, irritability, and hallucinations. Another study that included patients with left or right hemisphere stroke, probable AD, major depression, and elderly controls found no relationship between apathy and depression across the entire study sample.^92^ When analyzed by patient subgroup, there was a significant correlation between apathy and depression in those with left hemisphere stroke, probable AD, and major depression.^92^ In probable AD, there was a higher prevalence of apathy relative to depression.^92^ Similarly, of 131 individuals with possible or probable AD, 8% of the sample exhibited depression, and 60% manifested apathy (i.e., with symptoms of apathy at least 4–8 times per month).^70^ In a sample of 734 patients with mild AD, 9.4% had apathy only, 15.4% had depression only, and 32.4% had both apathy and depression.^12^ The most frequently occurring symptoms of depression were fatigue or loss of energy, decreased positive affect or pleasure in response to social contracts/activities, and psychomotor agitation/retardation; the most frequently occurring symptom of apathy was loss of/diminished goal‐directed cognitive activity. Finally, a recent review noted that people with apathy may tend toward passive/compliant behavior, lack suicidal ideation, and do not typically present with anxiety, rumination, or vegetative symptoms. People with depression were often reported as pessimistic, avoidant of socialization, possibly suicidal, and exhibited anxiety, rumination, and vegetative symptoms (i.e., poor sleep, loss of appetite, and weight loss).^10^

Taken together, these characterizations help inform differences and similarities in patient presentation between apathy and depression in those with NCDs. They also show features that are common across both syndromes.

## 4 DIFFERENTIATING APATHY FROM DEPRESSION REGARDING PROGNOSTIC VALUE

An analysis of 4932 individuals with MCI found that those with both apathy and depression had the greatest risk of developing AD when compared with the study reference group of those with no neuropsychiatric symptoms (NPS). When assessing risk of developing AD among individuals with apathy alone, or depression alone, only apathy was associated with a significantly greater risk for incident AD compared to no NPS.^18^ While depression is considered a risk factor for dementia, a substantial body of newer research suggests that late‐onset depression is a better marker of dementia risk than long‐standing depression, with the late‐onset depression representing prodromal symptoms of dementia in some individuals.^93^, ^94^, ^95^, ^96^ These findings emphasize the importance of and need for accurate diagnosis, especially in older individuals for whom the onset of NPS may be indicative of early stages of a NCD.

## 5 DISTINGUISHING APATHY AND DEPRESSION ON THE BASIS OF NEUROBIOLOGICAL SUBSTRATES AND BIOMARKERS

### 5.1 Pathophysiology of apathy and depression

Several neuroanatomical mechanisms are hypothesized to underlie apathy and depression. In elderly individuals without dementia, apathy has been associated with amyloid‐β (Aβ) pathology, indexed by the ratio of Aβ_42_ to t‐tau in the cerebrospinal fluid (CSF) and the burden of Aβ as shown by amyloid positron emission tomography (PET).^97^ A vascular contribution has been proposed, where apathy may result from destruction of limbic and reward pathways in the brain associated with small vessel disease (SVD).^98^, ^99^ Mechanisms associated with the presence of depression in NCD include vascular disease, hippocampal atrophy contributing to increased cortisol production, an increase in Aβ and tau, neuroinflammation, and reduction in neurotrophic factors.^100^ Although these studies suggest that similar neurobiological mechanisms may contribute to apathy and depression (e.g., Aβ pathology, vascular disease), several neuroimaging and biomarker studies have identified important differences as described below.

### 5.2 Neuroimaging investigations of the neurobiology of apathy and depression

The neuroanatomical correlates of both apathy and depression in dementia are well studied.^10^, ^101^, ^102^, ^103^, ^104^, ^105^, ^106^ Here, we provide an overview of investigations that have specifically focused on comparisons between apathy and depression with neuroimaging, providing insight into the unique neuroanatomical substrates of each syndrome.

An early review including data from individuals with various NCDs suggested that apathy results from dysfunction of frontal‐subcortical circuits.^107^ Moreover, reductions in gray matter density in medial frontal regions^108^ and various areas within the prefrontal cortex were associated with different aspects of apathy, including the dorsolateral prefrontal cortex (associated with the generation of cognitive plans or goals for action), the dorsomedial prefrontal cortex (associated with self‐initiated actions), and the orbital‐ventromedial prefrontal cortex (associated with emotional evaluation).^109^ Studies utilizing magnetic resonance imaging have demonstrated reduced integrity of frontal white matter in patients with apathy, which may lead to disruptions in frontal‐subcortical circuitry. A study of 79 patients with AD evaluated the relationship between white matter hyperintensities, apathy, and depression; 18% of patients were diagnosed with apathy alone, 19% with depression alone, 13% had both apathy and depression, and 50% had neither diagnosis.^110^ Analyses showed that patients with apathy had a larger volume of white matter hyperintensities in the frontal lobes compared with those without apathy, and those with depression had a larger volume of white matter hyperintensities in the right parietal lobe compared with those without depression.^110^

Although these findings support compromised frontal‐subcortical circuitry underlying apathy in NCDs, a recent report suggests that early temporal lobe changes may also play a role in producing apathy. In this analysis of patients with subjective cognitive decline and MCI, apathy (but not depression) assessed with the Mild Behavioral Impairment Checklist,^43^ was correlated with lower hippocampal volumes; there were no significant associations with frontal lobe volumes.^111^ Accordingly, changes in neuroanatomical regions affected early in AD may be associated with apathy, even in the absence of changes in frontal regions. In addition to the proposed role of the frontal‐subcortical circuitry in apathy, these results suggest that early medial temporal lobe involvement may contribute to the development of apathy in NCDs.

Comparing patients with AD plus apathy versus those without, 18F fluorodeoxyglucose PET showed reduced activity in bilateral anterior cingulate and medial orbitofrontal regions when analyses were corrected for age and cluster size.^112^ In a longitudinal evaluation of 953 elderly individuals without dementia followed for a median (standard deviation) of 43.5 (31.8) months, people with higher frontal Aβ deposition were at higher risk for developing apathy compared with those with lower Aβ deposition; these individuals also had a greater risk of cognitive decline.^97^ Furthermore, both tau^113^ and Aβ^114^ deposition in frontal regions have been associated with apathy in AD, independent of depression^113^ or gray matter volume,^114^ respectively.

An investigation comparing 2‐(1‐{6‐[(2‐[18F]fluoroethyl) (methyl)amino]‐2‐naphthyl}ylidene)malononitrile ([18^F^]FDDNP) protein binding in elderly individuals with late‐life depression without evidence of cognitive decline found that [18^F^]FDDNP aggregated protein binding in the anterior cingulate cortex was significantly related to apathy, but not depression, further implicating Aβ and tau in apathy.^115^ [18^F^]FDDNP binding, while associated with AD, has not been associated with late‐life depression.^116^

In FTD, a condition characterized by degeneration of the frontotemporal lobes,^117^ measurements of cortical thickness revealed dissociations between apathy and depression, with apathy severity correlated with thinning of regions in the right frontal and temporoparietal lobes.^118^ In contrast, an analysis comparing FTD patients with and without depression showed that those with depression had preserved cortical thickness in the right lateral and medial orbitofrontal cortices, right pars orbitalis, and right rostral anterior cingulate cortex compared to those without depression.^118^ These results highlight differing neuroanatomical correlates of apathy and depression.

Studies of individuals with cerebral SVD, manifested by confluent white matter lesions in periventricular or subcortical brain regions, have provided additional insight into the neuroanatomical differences of apathy and depression based on the integrity of white matter networks. An investigation of network disruption in 331 patients with SVD found that when compared with individuals without apathy, those with apathy demonstrated reduced connectivity in premotor and cingulate regions.^90^ In a whole‐brain analysis controlling for apathy, depression was not significantly associated with reduced connectivity in any region. In a study of 118 individuals with SVD, abnormalities of white matter integrity in anterior brain regions, as well as within parietal and temporal lobes (i.e., the bilateral anterior cingulum, corpus callosum, fornix, uncinate/inferior fronto‐occipital fasciculus, anterior thalamic radiation, anterior limbs of the external capsule) were related to apathy when controlling for depression; however, when controlling for apathy, there were no significant relationships between depression and compromised white matter integrity.^89^ These neuroanatomical correlates of apathy and depression emphasize the differing biology of the conditions.

### 5.3 Fluid biomarkers of apathy and depression

Previous research has investigated whether apathy and depression may be distinguishable on the basis of their fluid biomarker profiles. The ratio of Aβ_42_/Aβ_40_ was found to be significantly associated with persistent affective dysregulation (a composite of depression, anxiety, and elation/euphoria), but not with apathy, in a sample of memory clinic patients with MCI.^119^ Additionally, the role of Aβ_42_ in depression was shown in a cohort of individuals with late‐life depression and cognitive impairment compared with individuals with AD without depression.^116^ Those with late‐life depression and cognitive impairment had higher Aβ_42_ CSF levels and lower t‐tau and p‐tau CSF levels compared with those with AD without depression, suggesting that analysis of fluid biomarkers may aid in the diagnosis of AD versus late‐life depression. The relationship between Aβ_42_ and depression has not been a unanimous finding, nor have similar findings been demonstrated in apathy, as Aβ_42_ was not associated with depression or apathy in a sample of individuals with MCI.^120^ These results suggest that neither syndrome is strongly and consistently associated with biomarkers of AD and more information is required to understand these relationships.^10^ However, these findings may also reflect the inability of fluid‐based biomarkers to reflect regional differences as seen below.

A summary of findings distinguishing the neurobiological substrates of apathy and depression is found in Table 2. These findings highlight that just as apathy and depression may be distinguished by clinical presentation, each syndrome has, in some reports, been associated with distinct underlying pathophysiologic mechanisms, providing further support for the distinction between these neuropsychiatric conditions.

**TABLE 2:** Neuroanatomical correlates of apathy and depression.

Apathy	Depression
Cortical thickness	
Reduced cortical thickness in right frontal and temporoparietal regions^118^	Increased cortical thickness in right lateral and medial orbitofrontal cortices, right pars orbitalis, and right rostral anterior cingulate^118^
White matter networks	
Larger volumes of frontal white matter hyperintensities^110^	Larger volume of white matter hyperintensities in right parietal lobe^110^
Reduced white matter connectivity in premotor and cingulate regions^90^; white matter integrity in temporoparietal regions^89^	Not significantly correlated with reduced regional connectivity or white matter integrity^89^, ^90^
Hippocampal volume	
Correlated with lower hippocampal volume^111^	No relationship with hippocampal volume^111^
PET and fluid biomarkers	
Frontal Aβ deposition associated with risk of developing apathy^97^; associated with tau^113^ and Aβ^114^ deposition in frontal regions	Associated with the ratio of Aβ_42_/Aβ_40_ in plasma^119^

Abbreviation: PET, positron emission tomography.

## 6 DISTINGUISHING APATHY FROM DEPRESSION ON THE BASIS OF TREATMENT APPROACHES

Many drugs have been approved for the indication of depression, although none are approved specifically for depression in any NCD. There are currently no drugs indicated for apathy, resulting in an unmet need for this indication. Several neurotransmitter systems are thought to be involved in apathy (cholinergic, dopaminergic, serotonergic, noradrenergic, gamma‐aminobutyric acid‐ergic) and depression (serotonergic, noradrenergic, dopaminergic), with each of these potentially serving as a treatment target.^121^, ^122^

The American Psychiatric Association guidelines recommend a trial of antidepressants to treat patients with clinically significant depressed mood and dementia, with selective serotonin reuptake inhibitors (SSRIs) preferred over other types of antidepressants.^123^, ^124^ These agents do not address apathy, and a recent systematic review of pharmacological interventions for apathy in patients with AD found little evidence to support the use of antidepressants in patients with apathy.^121^ Despite lack of efficacy, antidepressant use is common in those with apathy. One cross‐sectional analysis of 684 community‐dwelling individuals with AD revealed that approximately one‐third of patients with apathy and no depression at inclusion were being treated with an antidepressant.^125^

Accurately distinguishing between apathy and depression is important, as the use of SSRIs may be associated with increased rates of apathy. In elderly adults without dementia, SSRI use significantly predicted apathy, with SSRIs associated with higher rates of apathy compared with non‐SSRI antidepressants.^87^ A cross‐sectional, retrospective study of 125 patients indicated that those treated with SSRIs had significantly higher scores on the AES‐Clinician rating compared with those not treated with SSRIs.^126^ Of patients treated with SSRIs, 92% had clinically significant apathy (i.e., AES score >30) versus 61% for those not treated with SSRIs. An SSRI‐induced apathy syndrome has been reported in a series of case reports from the general adult population.^127^ Although the precise mechanisms are not well understood, SSRIs cause elevated levels of serotonin that may lead to alterations in dopamine levels and downregulation of dopaminergic receptors, in turn leading to downregulation of responses to both rewarding and aversive stimuli and the emergence of apathy.^128^, ^129^

Although it is beyond the scope of the current review to discuss investigational treatments for apathy (e.g., the Apathy in Dementia Methylphenidate Trials [ADMET and ADMET‐2] of methylphenidate^130^, ^131^), previous evidence demonstrates that treatments for depression (e.g., SSRIs, selective serotonin norepinephrine reuptake inhibitors, bupropion) are largely ineffective for apathy, providing further mechanistic evidence for the differentiation of apathy from depression.^132^, ^133^

### 6.1 Behavioral approaches to treating apathy and depression

Considering that apathy and depression have both shared and unique symptoms, these syndromes may have similar or differing responses to nonpharmacological interventions depending on the target manifestation. Where symptoms overlap, approaches may also overlap. Several nonpharmacological approaches to treating apathy have been recommended, including exercise, music therapy, multisensory stimulation, pet therapy, and the use of digital therapies.^134^, ^135^ For depression, music therapy and psychological treatment (e.g., cognitive behavioral therapy) have been shown to significantly improve symptoms in those with dementia.^136^ Although nonpharmacological approaches may be beneficial for both apathy and depression, more research is needed regarding the use of these treatments. Additionally, each syndrome, given its unique symptoms, should be measured with syndrome‐specific outcomes.^137^

### 6.2 Brain stimulation approaches to apathy and depression

Transcranial magnetic stimulation (TMS) and transcranial direct current stimulation (tDCS) are additional nonpharmacological approaches utilizing noninvasive brain stimulation as possible treatments for apathy and depression. These methods are hypothesized to increase plasticity in regions thought to underlie apathy and depression to improve symptoms but have shown mixed efficacy in the treatment of apathy or depression in patients with dementia or MCI. A phase 2, single‐center, double‐blind, sham‐controlled, parallel‐group clinical trial of tDCS in 40 patients with moderate AD found that six sessions of anodal stimulation of the left dorsolateral prefrontal cortex did not decrease apathy scores or depressive symptoms.^138^ Another randomized, double‐blind, placebo‐controlled study of 4 weeks of cognitive training and adjunct repetitive TMS (rTMS) to the left dorsolateral prefrontal cortex in 50 individuals with MCI and AD showed no significant improvement in scores on the GDS versus sham stimulation.^139^

In contrast, a trial of 56 patients with probable AD found a significant improvement in depression scores for both the sham and rTMS cohorts following 2 weeks of stimulation to the temporoparietal cortex.^140^ A separate 5‐day trial of rTMS to the dorsolateral prefrontal cortex in 45 patients with probable AD found improvements in the GDS and measures of cognition and function (Mini Mental Status Exam, Instrumental Activities of Daily Living) when compared with sham therapy; improvements persisted for up to 3 months post‐treatment.^141^ Reductions in depressive symptoms were hypothesized to have been driven by a possible increase in dopamine following rTMS treatment. Additionally, a study that used rTMS over the left dorsolateral prefrontal cortex found that rTMS was effective in improving apathy and cognition in patients with AD.^142^ In this study, 20 patients with AD received 20 sessions of either rTMS (*n* = 9) or sham stimulation (*n* = 11); those who received rTMS demonstrated a statistically significant and clinically meaningful reduction in apathy scores, while those who received sham stimulation showed no improvement. The authors interpreted this result as an effect of rTMS on the dorsolateral prefrontal cortex, where enhanced dopamine transmission may contribute to reduction of apathy.

The lack of consistent efficacy for improving apathy and depression across investigations of noninvasive brain stimulation may be attributed to study methodologies, including the stimulation parameters utilized, diagnostic criteria for the NCD or the behavioral syndrome, or a study's sample size.^143^ Considering the promising evidence from some trials, future research with noninvasive brain stimulation for treating apathy and depression is warranted. Trials demonstrating efficacy in improving apathy and depression may provide further evidence to support the distinct neuroanatomical substrates of apathy and depression in this patient population.

## 7 SUMMARY AND CONCLUSIONS

The projected increase in the world's aging population and subsequent increase in neurodegenerative conditions make proper evaluation and treatment of apathy an urgent priority for the management of patients with dementia. Although apathy and depression may be difficult to distinguish within the setting of NCDs, the research reviewed here emphasizes that these two syndromes are distinct and may be distinguished by behavioral features, underlying neurobiology (as reflected in brain imaging), and responses to specific treatments. Accurate diagnosis is necessary for appropriate patient management, and recognition of apathy as a unique syndrome potentially benefiting from targeted pharmacologic and/or nonpharmacologic therapy is important for advancing clinical trials and treatment interventions for apathy. Future research is warranted to characterize additional methods for differentiating apathy from depression, and to evaluate novel, targeted treatments for apathy that may help to alleviate the burden for patients and their caregivers.

## CONFLICT OF INTEREST STATEMENT

**Krista L. Lanctôt**: Consultant or Advisory Board member for BioXcel Therapeutics, Cerevel Therapeutics, Eisai Co, Ltd, GW Pharmaceuticals, ICG Pharma, Kondor Pharma, H Lundbeck A/S, Merck Sharp Dohme, Novo Nordisk, Praxis Therapeutics; **Krista L. Lanctôt** is supported by the Alzheimer's Disease Drug Discovery Foundation (ADDF) and the Bernick Chair in Geriatric Psychopharmacology; **Kritleen K. Bawa** has nothing to disclose; **Jeffrey L. Cummings**: Consultant to Acadia, Alkahast, AlphaCognition, AriBio, Avanir, Axsome, Behren Therapeutics, Biogen, Biohaven, Cassava, Cerecin, Cortexyme, Diadem, EIP Pharma, Eisai, GemVax, Genentech, Green Valley, Grifols, Janssen, LSP, Merck, NervGen, Novo Nordisk, Oligomerix, Ono, Otsuka, PRODEO, Prothena, ReMYND, Renew, Resverlogix, Roche, Signant Health, Suven, United Neuroscience, and Unlearn AI pharmaceutical, assessment, and investment companies. **Jeffrey L. Cummings** is supported by NIGMS grant P20GM109025; NINDS grant U01NS093334; NIA grant R01AG053798; NIA grant P20AG068053; NIA grant R35AG71476; Alzheimer's Disease Drug Discovery Foundation (ADDF); and the Joy Chambers‐Grundy Endowment; **Masud Husain**: Consultant for Lilly, Otsuka Pharmaceuticals, and Sumitomo Dainippon Pharma; **Moyra E. Mortby** has nothing to disclose; **Philippe Robert** has nothing to disclose; **Zahinoor Ismail**: has served at scientific advisory boards and/or as a consultant to Lundbeck/Otsuka. His institution has received funds from Acadia, Biogen, and Roche.

## Data Availability

Data sharing is not applicable to this article as no new data were created or analyzed in this study.
